# Avaliação de Várias Vias Fisiopatológicas no Prognóstico da Insuficiência Cardíaca com Fração de Ejeção Reduzida: Vendo Além do Coração

**DOI:** 10.36660/abc.20220314

**Published:** 2022-06-06

**Authors:** João Marcos Barbosa-Ferreira, Andreza Araújo de Oliveira

**Affiliations:** 1 Universidade Nilton Lins Manaus AM Brasil Universidade Nilton Lins, Manaus, AM - Brasil

**Keywords:** Insuficiência Cardíaca, Prognóstico, Biomarcadores

O manuscrito “Escore albumina-bilirrubina para predizer desfechos em pacientes com cardiomiopatia dilatada idiopática” fornece uma maneira importante de avaliar o prognóstico de pacientes com cardiomiopatia dilatada (CMD), demonstrando uma maior taxa de eventos clínicos adversos maiores (ECAMs) em pacientes com pontuação mais alta. Além disso, esse escore funciona como um preditor independente de mortalidade em longo prazo.^1^

A Insuficiência Cardíaca com Fração de Ejeção Reduzida (ICFEr), muitas vezes caracterizada como CMD, tem seus aspectos fisiopatológicos intimamente relacionados à sua terapêutica e prognóstico. O estudo da fisiopatologia da ICFEr baseia-se nas alterações hemodinâmicas cardiovasculares e a intensa ativação neuro-humoral (principalmente do sistema nervoso autônomo, sistema renina-angiotensina-aldosterona e sistema peptídeos natriuréticos). Esses aspectos fisiopatológicos são amplamente utilizados na terapia e avaliação prognóstica de pacientes com ICFEr.^2^

Em relação ao prognóstico, as variáveis mais estudadas estão relacionadas a aspectos dessa fisiopatologia ou seus aspectos clínicos, como fração de ejeção, remodelamento cardíaco, dosagem de catecolaminas, capacidade funcional, consumo máximo de oxigênio, dosagem de peptídeo natriurético, classe funcional, ultrassonografia pulmonar, entre outros marcadores.^3-5^

Mais recentemente, outras vias fisiopatológicas têm sido cada vez mais estudadas e incorporadas ao tratamento de pacientes com ICFEr. Um exemplo disso é o estudo das alterações no metabolismo da glicose e seu tratamento neste grupo de pacientes. Portanto, a avaliação de outras vias metabólicas ou o envolvimento de outros órgãos e sistemas em pacientes com ICFEr é um aspecto importante a ser estudado quanto ao prognóstico desses pacientes.^6^

Rahimi et al. publicaram uma revisão sistemática em que as principais variáveis prognósticas estavam relacionadas aos aspectos clínico-epidemiológicos ou aos aspectos fisiopatológicos mais tradicionalmente estudados, como idade, sexo, função renal, pressão arterial e fração de ejeção, classe funcional, capacidade funcional e níveis de peptídeos natriuréticos. No entanto, outros parâmetros como diabetes, peso ou índice de massa corporal também foram associados a um pior prognóstico.^3^

Outros parâmetros não diretamente relacionados ao coração também foram associados a pior prognóstico na ICFEr. Alatas et al. demonstraram em uma análise multivariada que a microalbuminúria prediz a mortalidade intra-hospitalar em pacientes com ICFEr e fração de ejeção de médio alcance (ICFEm), mas não em fração de ejeção preservada (ICFEp).^7^ A anemia e o metabolismo do ferro foram extensivamente estudados para melhorar os sintomas e a qualidade da vida e avaliação prognóstica de pacientes com IC.^8^ Além disso, Tavares et al. observaram associação entre caquexia e desnutrição com mortalidade em pacientes com cardiopatia chagásica crônica, achados também encontrados em outras etiologias.^9^

Portanto, um maior conhecimento da importância do envolvimento de outros órgãos em pacientes com IC pode melhorar a avaliação geral desses pacientes. Nesse contexto, a disfunção hepática avaliada pelo escore albumina-bilirrubina é útil para uma avaliação prognóstica mais completa. Outros estudos demonstraram a importância da disfunção hepática também em pacientes com insuficiência cardíaca aguda.^10,11^

Sabemos que a ICFEr vem apresentando uma melhora substancial nas curvas de mortalidade ao longo dos anos, mas permanece com altas taxas de mortalidade, principalmente entre 5 e 10 anos.^12,13^ Novas formas de avaliação, incluindo o acometimento de outros órgãos e sistemas e/ou mesmo a avaliação genética, podem contribuir para uma melhora ainda maior dessas curvas de mortalidade por meio de uma melhor terapia e avaliação prognóstica.^14^
