# Supplementary material for: Avaliação de Várias Vias Fisiopatológicas no Prognóstico da Insuficiência Cardíaca com Fração de Ejeção Reduzida: Vendo Além do Coração
Source: Arq Bras Cardiol. 2022 Jun 6;118(6):1116–7. [Article in Portuguese] doi: 10.36660/abc.20220314 (PMC9345160; doi:10.36660/abc.20220314)
Supplement: Translation

# Translation: Evaluation of Various Pathophysiological Pathways in the Prognosis of Heart Failure with Reduced Ejection Fraction: Seeing Beyond the Heart

**Authors:** João Marcos Barbosa-Ferreira, Andreza Araújo de Oliveira

**Affiliations:** 1 Manaus AM Brazil Universidade Nilton Lins, Manaus, AM - Brazil

**Keywords:** Heart Failure, Prognosis, Biomarkers

The manuscript “Albumin-bilirubin score to predict outcomes in patients with idiopathic dilated cardiomyopathy” provides an important way to assess the prognosis of patients with dilated cardiomyopathy (DCM) by demonstrating a higher rate of major adverse clinical events (MACEs) in patients with a higher score. Furthermore, this score works as an independent predictor of long-term mortality.^1^

Heart Failure with reduced ejection fraction (HFrEF), often characterized as DCM, has its pathophysiological aspects closely related to its therapy and prognosis. The study of the pathophysiology of HFrEF is based on cardiovascular hemodynamic alterations and intense neurohumoral activation (mainly of the autonomic nervous system, renin-angiotensin-aldosterone system and natriuretic peptides system). These pathophysiological aspects are widely used in the therapy and prognostic assessment of patients with HFrEF.^2^

Regarding prognosis, the most studied variables are related to aspects of this pathophysiology or its clinical aspects, such as ejection fraction, cardiac remodeling, catecholamine dosage, functional capacity, maximum oxygen consumption, natriuretic peptide dosage, functional class, pulmonary ultrasound, among other markers.^3-5^

More recently, other pathophysiological pathways have been increasingly studied and incorporated into the treatment of patients with HFrEF. An example of this is the study of changes in glucose metabolism and its treatment in this group of patients. Therefore, the evaluation of other metabolic pathways or the involvement of other organs and systems in patients with HFrEF is an important aspect to be studied regarding the prognosis of these patients.^6^

Rahimi et al. published a systematic review in which the main prognostic variables were related to clinical-epidemiological aspects or the most traditionally studied aspects of pathophysiology, such as age, sex, renal function, blood pressure, and ejection fraction, functional class, functional capacity and levels of natriuretic peptides. However, other parameters such as diabetes, weight or body mass index were also associated with a worse prognosis.^3^

Other parameters not directly related to the heart have also been associated with a worse prognosis in HFrEF. Alatas et al. demonstrated in a multivariate analysis that microalbuminuria predicted in-hospital mortality in patients with HFrEF and mid-range ejection fraction (HFmrEF) but not in preserved ejection fraction (HFpEF).^7^ Anemia and iron metabolism have been extensively studied to improve symptoms and quality of life and the prognostic assessment of patients with HF.^8^ In addition, Tavares et al. observed an association between cachexia and malnutrition with mortality in patients with chronic Chagas’ heart disease, findings also found in other etiologies.^9^

Therefore, greater knowledge of the importance of the involvement of other organs in patients with HF may improve the general assessment of these patients. In this context, liver dysfunction assessed by the Albumin-bilirubin score is useful for a more complete prognostic assessment. Other studies demonstrated the importance of the liver dysfunction also in patients with acute heart failure.^10,11^

We know that HFrEF has shown a substantial improvement in mortality curves over the years, but it remains with high mortality rates, especially between 5 and 10 years.^12,13^ New forms of evaluation, including the involvement of other organs and systems and/or even genetic evaluation, may contribute to an even greater improvement in these mortality curves through improved therapy and prognostic assessment.^14^
